# Tetracenomycin X Exerts Antitumour Activity in Lung Cancer Cells through the Downregulation of Cyclin D1

**DOI:** 10.3390/md17010063

**Published:** 2019-01-18

**Authors:** Xinran Qiao, Maoluo Gan, Chen Wang, Bin Liu, Yue Shang, Yi Li, Shuzhen Chen

**Affiliations:** Institute of Medicinal Biotechnology, Chinese Academy of Medical Science & Peking Union Medical College, 1# Tiantan Xili, Chong Wen District, Beijing 100050, China; qxinran_yss@126.com (X.Q.); ganml@imb.pumc.edu.cn (M.G.); wangc1014@163.com (C.W.); bin0629bin@163.com (B.L.); shyue5775@163.com (Y.S.); liyi0108@163.com (Y.L.)

**Keywords:** tetracenomycin X, cell cycle arrest, cyclin D1, proteasomal degradation, p38, c-JUN

## Abstract

Tetracenomycin X (Tcm X) has been reported to have antitumour activity in various cancers, but there have not been any studies on its activity with respect to lung cancer to date. Therefore, this study aims to investigate the anti-lung cancer activity of Tcm X. In this study, we found that tetracenomycin X showed antitumour activity in vivo and selectively inhibited the proliferation of lung cancer cells without influencing lung fibroblasts. In addition, apoptosis and autophagy did not contribute to the antitumour activity. Tetracenomycin X exerts antitumour activity through cell cycle arrest induced by the downregulation of cyclin D1. To explore the specific mechanism, we found that tetracenomycin X directly induced cyclin D1 proteasomal degradation and indirectly downregulated cyclin D1 via the activation of p38 and c-JUN proteins. All these findings were explored for the first time, which indicated that tetracenomycin X may be a powerful antimitotic class of anticancer drug candidates for the treatment of lung cancer in the future.

## 1. Introduction

Lung cancer is one of the fastest growing malignancies with respect to morbidity and mortality worldwide. It is estimated that lung cancer caused 1.59 million deaths across the world in 2012, and the number of lung cancer deaths is forecasted to increase to 3 million in 2035 [1,2]. Traditional radiotherapy and chemotherapy do not have a successful impact on many lung cancer patients. Recent studies have found that there was only a 16% survival rate in lung cancer patients over the last five years [3,4]. Therefore, new drugs are urgently needed to treat lung cancer.

Tetracenomycinsare, a discrete group of aromatic polyketide antibiotics produced by the *Streptomyces* and *Nocardia* species, shows antibacterial activities mainly against Gram-positive bacteria and moderate antitumour activities [5,6]. The representative members of this group of antibiotics consist of tetracenomycins C and X and elloramycins A–F. As part of our screening program for new antibiotics from marine-derived microorganisms, tetracenomycin X with a high yield (31.8 mg/L), together with the novel *seco*-tetracenomycin analogues, saccharothrixones A–I, from the marine-derived rare actinomycete *Saccharothrix* sp. 10-10 were isolated [7,8,9]. In the previous work, tetracenomycin X was found to show significant in vitro cytotoxic activities in leukaemia and liver and breast cancer cell lines [7,9]. Although tetracenomycins showed cytotoxic activities in many kinds of cancer cells, there have been few studies that have investigated their in vivo activity. To the best of our knowledge, the only member reported to have in vivo antitumour activity was tetracenomycin C, which displayed antitumour effects against leukaemia cells (P388) in mice [10]. However, their in vivo activities against lung cancer and antitumour mechanisms have not been investigated thus far. This has encouraged us to further explore the anti-lung cancer and antitumour mechanisms of tetracenomycins.

In the current study, we examine the antitumour activity of tetracenomycin X in lung cancer cells and further explored its anticancer mechanisms.

## 2. Results

### 2.1. Tetracenomycin X Exerts Antitumour Activity in H460 Xenografts in BALB/c Nude Mice

**Figure 1:**
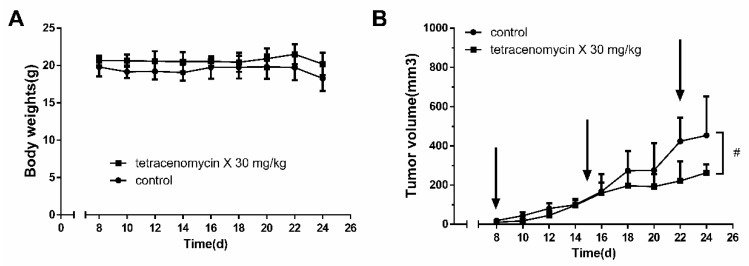
Tetracenomycin X exerts antitumour activity in H460 xenografts in BALB/c nude mice. (**A**) The body weight of H460 xenograft-bearing nude mice (n = 6). (**B**) The volume of H460 xenografts in nude mice (n = 6). ^#^ *p* < 0.05 compared with the control group.

Because there have been few studies investigating the antitumour activity of tetracenomycin X in vivo, we first detected its antitumour activity in nude mice. As shown in Figure 1A, there were no deaths or significant weight changes in the two study groups, which suggested that the dose of tetracenomycin X was tolerated. Compared to the control group, tetracenomycin X significantly inhibited the growth of H460 xenografts (Figure 1B). The antitumour rate of the tetracenomycin X group was 42%.

### 2.2. Tetracenomycin X Selectively Inhibits Human Lung Cancer Cell Proliferation

**Figure 2:**
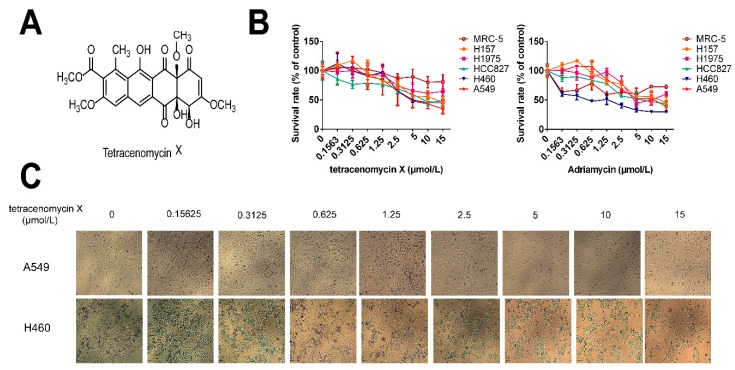
Tetracenomycin X selectively inhibits the cell proliferation of lung cancer cells. (**A**) The structure of tetracenomycin X. (**B**) The proliferative activity of the five lung cancer cells and lung fibroblasts after being treated with tetracenomycin X and Adriamycin (0.1563, 0.3125, 0.625, 1.25, 2.5, 5, 10 and 15 µmol/L) for 24 h was assessed by sulforhodamine B (SRB) assay. The survival rates were calculated as a ratio compared with the control group (untreated cells). The values represent the mean ± SD of three independent samples. Each experiment was repeated three times under each condition. (**C**) The cell morphology of the A549 and H460 cells under a 4 *0.1 microscope after treatment with tetracenomycin X for 24 h.

Tetracenomycin X, an aromatic polyketide antibiotic, was identified from the marine-derived actinomycete *Saccharothrix* sp. 10-10 by Professor Maoluo Gan at our institute [9]. Its structure is shown in Figure 2A. As tetracenomycin X is similar to Adriamycin in structure, Adriamycin was selected as a positive drug with which to compare the antitumour activity of tetracenomycin X. The anti-proliferative activity of tetraccenomycin X and Adriamycin were tested against five lung cancer cells (H157, H1975, HCC827, H460 and A549) and one lung fibroblasts (MRC-5). As shown in Figure 2B, tetracenomycin X hardly inhibited the proliferation of MRC-5 compared with the other lung cancer cells, whereas it significantly inhibited the growth of the A549 cells and H460 cells in a dose-dependent manner among the five lung cancer cells. The half-inhibitory concentration (IC_50_) upon 24-h treatment was 6.41 ± 0.87 µmol/L and 5.42 ± 1.17 µmol/L in the A549 and H460 cells, respectively. Adriamycin, on the other hand, showed good anti-proliferation activity not only in the four types of lung cancer cells, but also in the normal lung MRC-5 fibroblasts. The IC_50_ upon 24-h treatment of Adriamycin was 7.58 ± 2.21 µmol/L and 0.60 ± 0.26 µmol/L in the A549 and H460 cells, respectively. It was obvious that tetracenomycin X selectively targeted lung cancer cells without inducing cytotoxicity in normal cells, but this advantage was not available to Adriamycin. Furthermore, we found that there was no change in the A549 and H460 cell morphology, while the cell density decreased clearly as the concentration of tetracenomycin X increased (Figure 2C).

### 2.3. The Antitumour Activity of Tetracenomycin X Is Independent of Apoptosis and Autophagy

**Figure 3:**
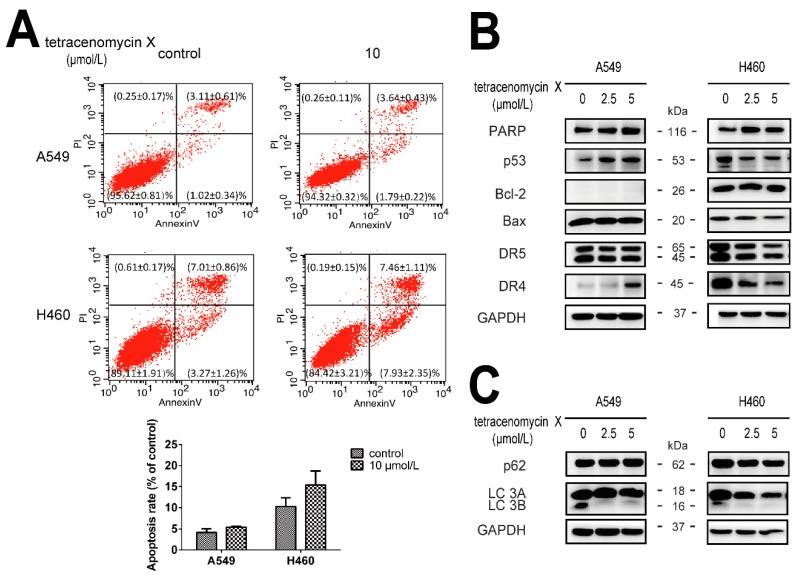
The antitumour activity of tetracenomycin X is independent of apoptosis and autophagy. (**A**,**C**) The A549 and H460 cells were treated with tetracenomycin X (2.5 and 5 µmol/L) for 8 h. The expression levels of the proteins were determined using western blotting. (**B**) The A549 and H460 cells were treated with tetracenomycin X (10 µmol/L) for 24 h. Apoptosis was detected by annexin V-FITC/PI staining and flow cytometry analysis.

Annexin V-FITC/PI (Annexin V-fluoresceine isothiocyanate/Propidium Iodide) staining and flow cytometry analysis were performed to detect apoptosis. As shown in Figure 3A, the apoptosis rate in the A549 cells was (4.13 ± 0.94)% in the control group and (5.43 ± 0.21)% in the tetracenomycin X (10 µmol/L) group. Similarly, the apoptosis rate in the H460 cells was (10.28 ± 2.05)% in the control group and (15.39 ± 3.34)% in the tetracenomycin X (10 µmol/L) group. The apoptosis rate was too low in the tetracenomycin X group in two cells. Furthermore, western blotting was used to examine the changes in the protein levels involved in the apoptosis (PARP (poly ADP-ribose polymerase), p53, DR4/5 (death receptor 4/5), Bcl-2 (B-cell lymphoma-2) and Bax (Bcl-2 Associated X Protein) and autophagy (p62 and LC-3B) pathways. As shown in Figure 3B, tetracenomycin X at a concentration of 2.5 or 5 µmol/L did not induce the cleavage of PARP in the A549 and H460 cells and increased the expression of p53 and DR4 in the A549 cells, which were decreased in the H460 cells. In addition, the expression of the DR5, Bcl-2 and Bax proteins also showed different changes in these two cells under the tetracenomycin X treatment. Interestingly, tetracenomycin X reduced the expression of the LC-3B protein but had no effect on the p62 protein (Figure 3C). Tetracenomycin X induced different changes or no change in the expression of the same protein in different cells, indicating that this protein was not the key effector protein in the antitumour activity of tetracenomycin X.

### 2.4. Tetracenomycin X Induces Cell Cycle Arrest in the G0/G1 Phase and Decreases the Expression Levels of Cell Cycle-Related Proteins in Lung Cancer Cells

**Figure 4:**
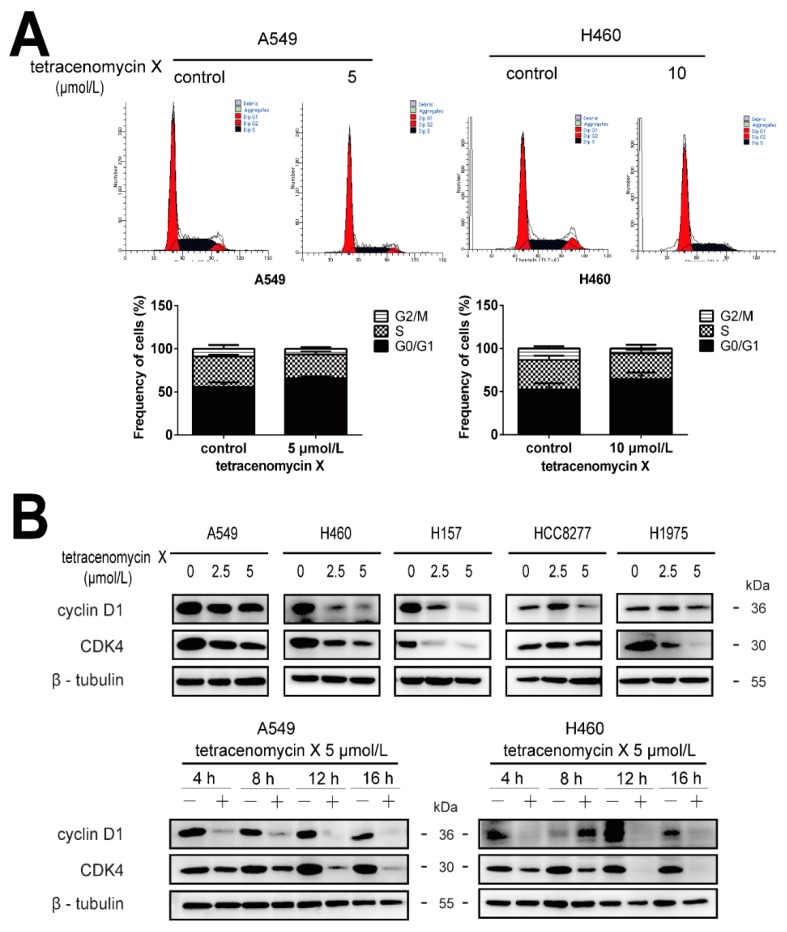
Tetracenomycin X-induced cell cycle arrest in the G0/G1 phase and decreased the expression levels of cell cycle-related proteins in the lung cancer cells. (**A**) the A549 and H460 cells were treated with tetracenomycin X (5 or 10 µmol/L) for 16 h. The cell cycle was detected by flow cytometry analysis. (**B**) The A549 and H460 cells were treated with tetracenomycin X for the indicated times or treated with various concentrations of tetracenomycin X for 8 h. The expression levels of proteins were determined using western blotting.

To elucidate the antitumour mechanism of tetracenomycin X in lung cancer cells, flow cytometry analysis was used to observe the cell cycle distribution in different groups. We found that the percentage of the G0/G1 phase in the control and tetracenomycin X (5 µmol/L) groups in the A549 cells was (55.51 ± 5.71)% and (65.94 ± 2.24)%, respectively. Additionally, the percentage of the G0/G1 phase in the H460 cells was (52.25 ± 7.38)% in the control group and (64.48 ± 7.66)% in the tetracenomycin X (10 µmol/L) group (Figure 4A). We next performed western blotting to assess the expression levels of the cell cycle-related proteins. As shown in Figure 4B, doses of tetracenomycin X at 2.5 and 5 µmol/L abated the levels of cyclin D1 and CDK4 (cyclin-dependent kinase 4) in the five lung cancer cells. Furthermore, tetracenomycin X (5 µmol/L) decreased the expression of cyclin D1 and CDK4 in 4–16 h in the A549 and H460 cells.

### 2.5. Tetracenomycin X Induces the Proteasomal Degradation of Cyclin D1

**Figure 5:**
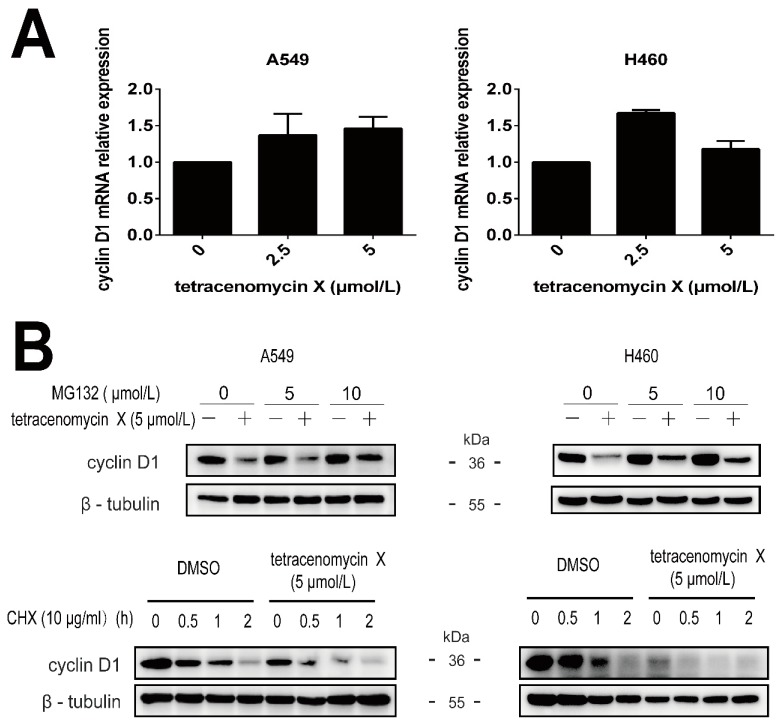
Tetracenomycin X induces the proteasomal degradation of cyclin D1. (**A**) RT-PCR analysis of the gene expression of cyclin D1 in the A549 cells and H460 cells. (**B**) The A549 cells and H460 cells were pre-treated with MG132 at the indicated concentration for 2 h and then co-treated with tetracenomycin X (5 µmol/L) for 12 h. The A549 cells and H460 cells were pre-treated with DMSO or tetracenomycin X (5 µmol/L) and then co-treated with 10 μg/mL of cycloheximide (CHX) for the indicated times.

To verify whether the downregulation of cyclin D1 by tetracenomycin X is mediated by suppression at the mRNA level or by proteasomal degradation, we first used real-time PCR to assess the mRNA level of cyclin D1 in the A549 cells and H460 cells. However, the cyclin D1 mRNA relative expression was increased under doses of the tetracenomycin X treatment (Figure 5A). Then, MG132 and cycloheximide (CHX) were used to investigate the proteasomal degradation of cyclin D1. MG132 is a proteasome inhibitor, and CHX is usually used to inhibit protein synthesis by inhibiting peptidyl transferase activity in eukaryotic cells [11]. As shown in Figure 5B, we found that MG132 at the concentration of 10 µmol/L attenuated the tetracenomycin X-mediated downregulation of cyclin D1, and the combination of tetracenomycin X and CHX enhanced the degradation of cyclin D1 compared with the CHX-added DMSO group. All of these indicated that tetracenomycin X induced the degradation of cyclin D1 through the proteasomal degradation pathway rather than through the suppression of the mRNA level.

### 2.6. Tetracenomycin X Decreases the Expression of Cyclin D1 by the Activation of p38 and c-JUN

**Figure 6:**
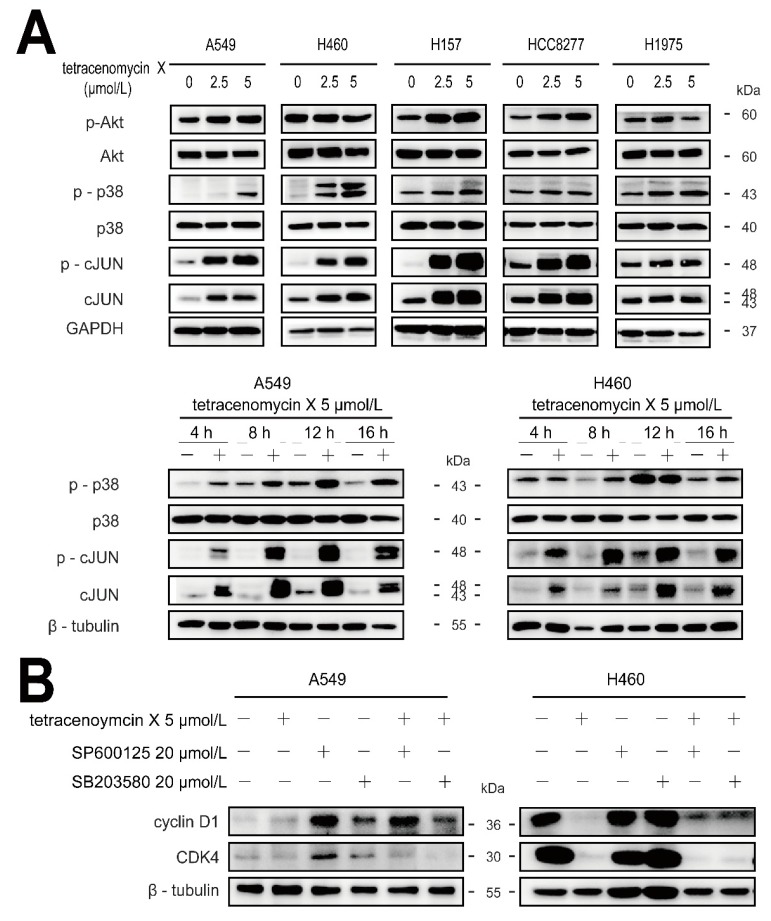
Tetracenomycin X decreases the expression of cyclin D1 by the activation of p38 and c-JUN. (**A**) The A549 and H460 cells were treated with tetracenomycin X for the indicated times or treated with various concentrations of tetracenomycin X for 8 h. The expression levels of the proteins were determined using western blotting. (**B**) The viability of the A5459 and H460 cells was determined after a 4-h pre-treatment with SP600125 or SB203580 and an 8-h treatment with tetracenomycin X. The expression levels of the proteins were determined using western blotting.

Studies have showed that the changes in the expression of cyclin D1 are associated with Erk1/2, p38 and Akt [12,13,14]. To further elucidate the antitumour mechanism of tetracenomycin X, we tested its impact on the MAPK and Akt signalling pathways. As shown in Figure 6A, tetracenomycin X activated the phosphorylation of Akt in the A549 cells but inactivated it in the H460 cells. We found that doses of tetracenomycin X elevated the phosphorylation levels of the p38 and c-JUN proteins in the five lung cancer cells, and tetracenomycin X (5 µmol/L) also active the p38 and c-JUN proteins in 4–16 h in the A549 and H460 cells. Accordingly, we used SP600125 (a JNK inhibitor) and SB203580 (a p38 MAPK inhibitor) in combination with tetracenomycin X to treat the A549 and H460 cells. The western blotting results showed that both the inhibitors reversed the tetracenomycin X-induced degradation of cyclin D1, while they did not increase the expression of CDK4 (Figure 6B).

## 3. Discussion

Tetracenomycins, a family of tetracyclic haphthacenequinones, have large anti-Gram-positive effects and significant antitumour activity. Tetracenomycin X, as a member of this family, was only reported to have antitumour activity in leukaemia cells, liver cancer cells and breast cancer cells [6,7,8,9,15,16]. This study is the first to show that tetracenomycin X possesses antitumour activity in H460 xenografts in BALB/c nude mice, so we studied the antitumour mechanism in depth. Experiments in vitro showed that tetracenomycin X selectively attenuates the proliferation of lung cancer cells without inhibiting the lung fibroblasts, while this inhibitory effect was significant in the A549 cells and H460 cells. At the same time, there was no obvious difference in the effect of Adriamycin on the anti-proliferation activity between the lung cancer cells and normal lung fibroblasts. Furthermore, few studies have described the antitumour mechanism of tetracenomycin X, so we needed to examine the cell death pathways one by one. First, we tested the changes in apoptosis and autophagy. The results showed that neither apoptosis nor autophagy was involved in the antitumour mechanism. Then, we performed flow cytometry analysis to observe cell cycle distribution. We finally found that tetracenomycin X could increase cell cycle arrest at the G0/G1 phase, which suggested that the induction of cell cycle arrest was the major antitumour mechanism in the lung cancer cells.

The cell cycle, which includes four phases (G1, S, G2 and M), is the basic process to guarantee cell proliferation. The four phases transition in sequence under exogenous and endogenous regulations [14]. Exogenous regulation is mainly caused by cytokines, and endogenous regulation is induced by the cyclin–CDK network. Cyclins and cyclin-dependent kinases (CDKs) are sensitive to oncogenic stimulation, and their excessive activation can make cancer cells grow strongly [17]. Cyclin D1 together with CDK4 plays an important role in promoting the cell cycle from the G0/G1 phase to the S phase. Accordingly, changes in the complex of cyclin D1 and CDK4 may be responsible for the tetracenomycin X-induced cell cycle arrest. As expected, decreases in cyclin D1 and CDK4 were found in the lung cancer cells after the tetracenomycin X treatment.

Recent studies have shown that cyclin D1 expression can be regulated through transcription and proteasomal degradation, and the proteasomal degradation of cyclin D1 has been thought to be an important antitumour pathway [18]. The current study shows that tetracenomycin X increases the mRNA level of cyclin D1, while MG132 attenuates tetracenomycin X-induced cyclin D1 downregulation and CHX enhances the degradation of cyclin D1 after tetracenomycin X treatment. These findings suggest that cyclin D1 proteasomal degradation may be one of the important molecular targets for the antitumour activity of tetracenomycin X.

It has been reported that cyclin expression is regulated by upstream kinases, such as p38, Akt and JNK [13,19]. Our results show that tetracenomycin X only activates the phosphorylation levels of p38 and c-JUN proteins in lung cancer cells, which leads to the downregulation of cyclin D1. It appears that p38 and c-JUN activation by tetracenomycin X, at least in part, contributes to tetracenomycin X-mediated cyclin D1 downregulation.

**Figure 7:**
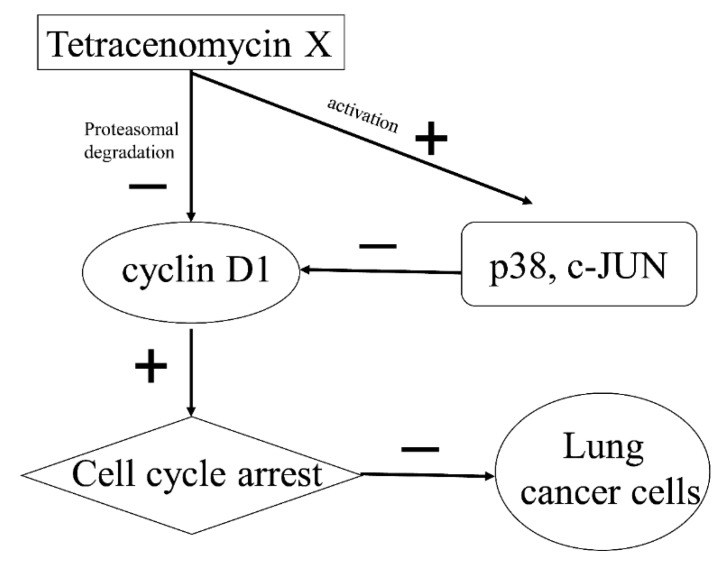
Schematic presentation of the antitumour mechanism. In lung cancer cells, tetracenomycin X directly induces cyclin D1 proteasomal degradation and indirectly downregulates cyclin D1 via the activation of p38 and c-JUN. The decrease in cyclin D1 results in cell cycle arrest at the G0/G1 phase and eventually inhibits the proliferation of lung cancer cells.

In conclusion, tetracenomycin X selectively exerts high anti-proliferation activity in lung cancer cells without inhibiting normal cells. Cyclin D1 proteasomal degradation-mediated cell cycle arrest at the G0/G1 phase may be the important antitumour mechanism of tetracenomycin X in lung cancer cells. In addition, p38 and c-JUN activation by tetracenomycin X partly results in cyclin D1 downregulation (Figure 7). Thus, tetracenomycin X may have the potential to become an antimitotic class of drugs against lung cancer.

## 4. Materials and Methods

### 4.1. Cell Culture

The human lung cancer A549, H460, H157, HCC827 and H1975 cell lines were kept in our laboratory and maintained in RPMI-1640 medium (ThermoFisher Scientific, Waltham, MA, USA) supplemented with 10% foetal bovine serum (Gibco, Carlsbad, CA, USA) and penicillin (100 U/mL)/streptomycin (100 µg/mL) (National China Pharmaceutical Inc., Beijing, China) at 37 °C in a 5% CO_2_ incubator. The human embryonic lung MRC-5 fibroblasts were purchased from National Institutes for Food and Drug Control (Beijing, China) and cultured in MEM-EBSS (Minimum Essential Medium-Earle’s Balanced Salts Solution) medium supplemented with 10% bovine serum and penicillin (100 U/mL)/streptomycin (100 µg/mL).

### 4.2. Reagents and Antibodies

Tetracenomycin X (Tcm X) was isolated from the marine-derived actinomycete *Saccharothrix* sp. 10-10 by Professor Maoluo Gan at our institute. The molecular weight of Tcm X was 486 Da, and it was dissolved in dimethyl sulfoxide (DMSO). The anti-p53, anti-PARP, anti-Bcl-2, anti-Bax, anti-p38 MAPK, anti-phospho-p38 MAPK, anti-cJUN, anti-phospho-cJUN, anti-cyclin D1 and anti-CDK4 antibodies were purchased from Cell Signaling Technology (Danvers, MA, USA). The anti-LC3B antibody was obtained from Sigma (St. Louis, MO, USA). The anti-Human CD261 Azide Free (DR4) antibody was obtained from Diaclone (Besancon, France), and the DR5 antibody was purchased from ProSci (San Diego, CA, USA). β-Actin (6G3), β-tubulin and GAPDH (glyceraldehyde-3-phosphate dehydrogenase) (1C4) mouse monoclonal antibodies were obtained from AmeriBiopharma (Wulmington, DE, USA). For the second antibodies, peroxidase-conjugated affiniPure goat anti-mouse IgG (H + L) and anti-rabbit IgG (H + L) were obtained from ZSGB-BIO (Beijing, China). MG132, cycloheximide (CHX), SB203580 and SP600125 were purchased from Selleck.cn (Houston, TX, USA). Sulforhodamine B (SRB) was obtained from Sigma (St. Louis, MO, USA). Trichloroacetic acid (TCA) was obtained from Solarbio (Beijing, China).

### 4.3. Cell Survival Assay

A sulforhodamine B (SRB) method was used to detect cell survival. The tested cell lines were seeded in 96-well plates at 4 × 10^3^ cells/well in 100 µl of culture medium for 24 h. Different concentrations of tetracenomycin X were then added to the 96-well plates and incubated for 24 h. The SRB method was described previously [20]. The cell survival was calculated as the ratio of the experimental groups to control group.

### 4.4. Western Blot Analysis

The cells were seeded in 6-well plates and exposed to drugs for specific times. The preparation of cell samples and western blot analyses were described previously [20].

### 4.5. Annexin V-FITC/PI Staining

The tested cells were plated on 6-well plates overnight and treated with drugs for 24 h. Then, cell apoptosis was examined by an Annexin V-FITC/PI apoptosis detection kit (Bio Friend, Beijing, China), according to the manufacturer’s instructions. The Annexin V-FITC/PI staining was detected by flow cytometry analysis.

A schematic plot was applied to analyse the results, in which the lower left quadrant represented live cells, the lower right and upper right quadrants represented early and late apoptotic cells, respectively, and the upper left quadrant represented necrotic cells. The cell apoptosis rate was the sum of the percentages of the early and late apoptotic cells.

### 4.6. Cell Cycle Analysis

The A549 cells and H460 cells were plated on 6-well plates and grown overnight. Then, the cells were treated with tetracenomycin X for 16 h. The cell cycle was determined using a PI (including RNase A) cell cycle detection kit (Bio Friend, Beijing, China) according to the manufacturer’s instructions and then analysed by a flow cytometer COULTER EPICS XL (Beckman Coulter, Inc., Brea, CA, USA).

### 4.7. Reverse Transcriptase-Polymerase Chain Reaction

The A549 cells were plated on 6-well plates and exposed to tetracenomycin X for 12 h. The total RNA was extracted by a RNA fast 200 Kit (FASTAGEN, Shanghai Fastagen Biotechnology, Shanghai, China), and 1 µg of RNA was used with a Prime Script^TM^ RT reagent kit with a gDNA Eraser (TaKaRa, Tokyo, Japan) according to the manufacturer’s instructions for cDNA synthesis. RT-PCR was carried out using a qPCR Mix Kit (TOYOBO, Osaka, Japan) with human primers for cyclin D1 and β-actin as follows: cyclin D1: forward 5′-aactacctggaccgcttcct-3′ and reverse 5′-ccacttgagcttgttcacca-3′, β-actin: forward 5′-agcgagcatcccccaaagtt-3′ and reverse 5′-gggcacgaaggctcatcatt-3′.

### 4.8. Lung Cancer Xenograft

The animal experiments were performed in the Experimental Animal Center of our institute and in accordance with all the relevant guidelines and regulations. First, the H460 cells (5 × 10^6^ cells in 200 µL of PBS) were injected into the right armpits of 6-week-old female BALB/c nude mice (Anikeeper, Beijing, China). Then, 21 days after inoculation, tumours were removed and cut into 2 mm × 2 mm × 2 mm prisms and transplanted into the right armpits of mice. On the 8th day, the mice were randomly divided into two groups: one was a control group and the other was a group given tetracenomycin X (30 mg/kg/day, dissolved in 10% alcohol) via intraperitoneal injection for 3 consecutive days in a week. The tumour volumes and body weights were monitored once every 2 days. The tumour volume was calculated by the following formula: *V* = ab^2^/2, where a represents the length and b represents the width. The animal experiment lasted for a total of 24 days.

### 4.9. Statistical Analysis

All the experiments were repeated at least 2 times. The data are expressed as the mean ± SD. The statistical analysis was performed via Student’s *t* test for the independent samples.
